# Impact of Molecular Syndromic Diagnosis of Severe Pneumonia in the Management of Critically Ill Patients

**DOI:** 10.1128/spectrum.01616-22

**Published:** 2022-09-26

**Authors:** Dimitra Stafylaki, Sofia Maraki, Katerina Vaporidi, Dimitrios Georgopoulos, Dimitrios P. Kontoyiannis, Diamantis P. Kofteridis, Georgios Chamilos

**Affiliations:** a Department of Clinical Microbiology and Microbial Pathogenesis, University Hospital of Heraklion, Heraklion, Crete, Greece; b ICU Department, University Hospital of Heraklion, Heraklion, Crete, Greece; c Internal Medicine Department, University Hospital of Heraklion, Heraklion, Crete, Greece; d Department of Infectious Diseases, The University of Texas, MD Anderson Cancer Center, Houston, Texas, USA; University of Mississippi Medical Center

**Keywords:** ICU, severe CAP, syndromic testing, VAP, multidrug resistance, pneumonia

## Abstract

The impact of syndromic molecular diagnosis in the management of nosocomial infections caused by multidrug-resistant (MDR) and extensively drug-resistant (XDR) pathogens has been incompletely characterized. We evaluated the performance of a molecular syndromic platform (BioFire FilmArray-Pneumonia plus Panel) in patients with pneumonia in the intensive care unit (ICU) of a University Hospital in Greece over a 2-year period. We evaluated 79 consecutive patients diagnosed with pneumonia in the ICU (2018–2020), including 55 patients with ventilator associated pneumonia (VAP). We included 40 control patients diagnosed with pneumonia in the ICU the year before the study (2017–2018). We identified 16 cases of VAP due to XDR bacterial pathogens. We found an excellent agreement (89.4% 76/85 reported results) between the results of syndromic platform and conventional cultures of tracheal aspirates. The molecular syndromic test significantly improved time to diagnosis versus conventional culture (3.5 h vs 72 h, *P < *0.0001), and identified new pathogens not detected by cultures in 49% of the cases. However, three cases of pneumonia with targets not included in the molecular platform, were not detected. Implementation of the molecular syndromic facilitated treatment modification from broad to narrow spectrum antimicrobial therapy, resulting in significant reductions in antibiotic consumption in the study group compared to the control group, without a negative impact in patient outcome. The implementation of syndromic molecular diagnosis in critically ill patients with pneumonia is associated with timely and improved diagnosis and has significant impact on reduction of antibiotic consumption.

**IMPORTANCE** The impact of syndromic molecular diagnosis in the management of nosocomial infections caused by MDR/XDR pathogens has been incompletely characterized. We evaluated the performance of a molecular syndromic platform (BioFire FilmArray -Pneumonia plus Panel) in 79 patients with pneumonia in the intensive care unit (ICU) of a University Hospital in Greece over a 2-year period (2018–2020) compared to 40 control patients diagnosed with pneumonia in the ICU the year before the study (2017–2018). Importantly, implementation of syndromic pneumonia panel improved time to diagnosis, identified new pathogens not detected by cultures in 49% of the cases and resulted in a significant reduction in antibiotic consumption compared to the year before initiation of the study without a negative impact in mortality of patients. Collectively, our study demonstrates the positive value of PCR syndromic testing in the management of pneumonia in ICUs high rates of MDR/XDR nosocomial pathogens.

## INTRODUCTION

Lower respiratory tract infections remain a leading cause of death worldwide (1). Furthermore, ventilator associated pneumonia (VAP) is a major cause of prolonged length of stay and poor outcome of patients in the Intensive Care Unit (ICU) (2). Timely initiation of appropriate antibiotic therapy is the most critical determinant of the outcome of patients with severe community acquired pneumonia (CAP), health care associated pneumonia (HAP) and VAP (3). Nonetheless, empirical selection of antimicrobial therapy and optimal management of severe CAP, HAP and VAP is challenging, because of the emergence of multi- and/or pan-drug resistant (MDR/PDR) nosocomial pathogens (4). In particular, infections by Carbapenem resistant Gram-negative bacterial pathogens (e.g., Klebsiella pneumoniae, Acinetobacter baumannii, Pseudomonas aeruginosa, and Enterobacter species) has become an epidemic in many European countries, including Greece (5). Therefore, empirical antibiotic therapy of severe CAP/HAP and VAP typically includes a combination of antibiotics, which is associated with increased toxicity and cost, and selection for antimicrobial resistance.

Molecular syndromic platforms are increasingly utilized in diagnosis of infectious diseases as a mean to improve etiological diagnosis and reduce antibiotic consumption through pathogen-guided empirical antimicrobial therapy (6–10). However, the value of these molecular diagnostics has not been previously tested in the setting of nosocomial infections due to multidrug resistant microbial pathogens. Herein, we evaluated a recently implemented molecular syndromic platform in the management of severe pneumonia in the ICU, in a setting of high rates of antimicrobial resistance. Of interest, implementation of syndromic pneumonia panel improved diagnosis and facilitated de-escalation of antimicrobial therapy, resulting in a significant reduction in antibiotic consumption, compared to the year before initiation of the study.

## RESULTS

We enrolled 79 consecutive critically ill patients diagnosed with different type of pneumonia (severe CAP/HAP or VAP) in the ICU and compared them with an historical control group of patients with pneumonia in the ICU during the year before initiation of the study. The demographics and clinical characteristics of both groups of patients are presented in Table 1. VAP comprised almost 2/3 of cases of pneumonia in both study groups (Table 1). There were no significant differences in demographic and clinical characteristics of the patients, including median age, co-morbidities, and severity of illness as evidenced by APACHE II and SOFA score (on day 1). Importantly, we found no significant differences in the outcome, including mean number of days of hospitalization in the ICU and mortality rates, between the two groups of patients (Table 1).

**TABLE 1:** Demographics, clinical characteristics and outcome of patients and historical controls

Characteristic	Control group (*n* = 40)	Syndromic PCR group (*n* = 79)	*P* value
Age	68 (20–86)	69 (22–87)	0.62
Sex			
Male Sex (%)	82.5 (*n* = 33)	67.1 (*n* = 53)	0.08
Type of Pneumonia			
VAP	57,5% (*n* = 23)	69,62% (*n* = 55)	0.22
Apache II	26 (10–41) ± 6.5	26 (7–45) ± 7.8	0.78
SOFA	11 (6–14) ± 2.8	11 (7–21) ± 2.9	0.77
Days of Hospitalization	17 (3–120) ± 19.63	17 (3–54) ± 11.4	0.31
Outcome			
Improvement	67.5% (*n* = 27)	78.48% (*n* = 62)	0.26
No change	12.5% (*n* = 5)	1.27% (*n* = 1)	0.02
Death	20% (*n* = 8)	20.25% (*n* = 16)	>0.99
Underlying disease			
COPD	20% (*n* = 8)	21.52% (*n* = 17)	>0.99
Arterial Hypertension	42.5% (*n* = 17)	56.96% (*n* = 45)	0.17
Diabetes Mellitus	22.5% (*n* = 9)	29.11% (*n* = 23)	0.52
Heart Failure	20% (*n* = 8)	31.65% (*n* = 25)	0.2
Liver Disease/Failure	7.5% (*n* = 3)	5.06% (*n* = 4)	0.69
Renal Disease/Failure	7,5% (*n* = 3)	15,19% (*n* = 12)	0.38
Hematological Diseases	0	5.06% (*n* = 4)	0.3
Cancer	7.5% (*n* = 3)	10.13% (*n* = 8)	0.75
Neurological Diseases	22.5% (*n* = 9)	15.19% (*n* = 12)	0.32
Drug Abuse	0	1.27% (*n* = 1)	>0.99
Alcohol	5% (*n* = 2)	2.53% (*n* = 2)	0.6
Obesity	5% (*n* = 2)	1,27% (*n* = 1)	0.26
No other comorbidity	30% (*n* = 12)	10.13% (*n* = 8)	0.0092
HIV	2.5% (*n* = 1)	0	0.34
Unknown	0	3.80% (*n* = 3)	0.55
Other immunodeficiency	0	5.06% (*n* = 4)	0.3
Bed-days	14 (2–14) ± 2.83	9 (1–14)+4.13	0.001
Time to diagnosis	72 (10.88–169.07) ± 38.26	3.5 (2.92–4.2) ± 0.3	<0.0001

**FIG 1:**
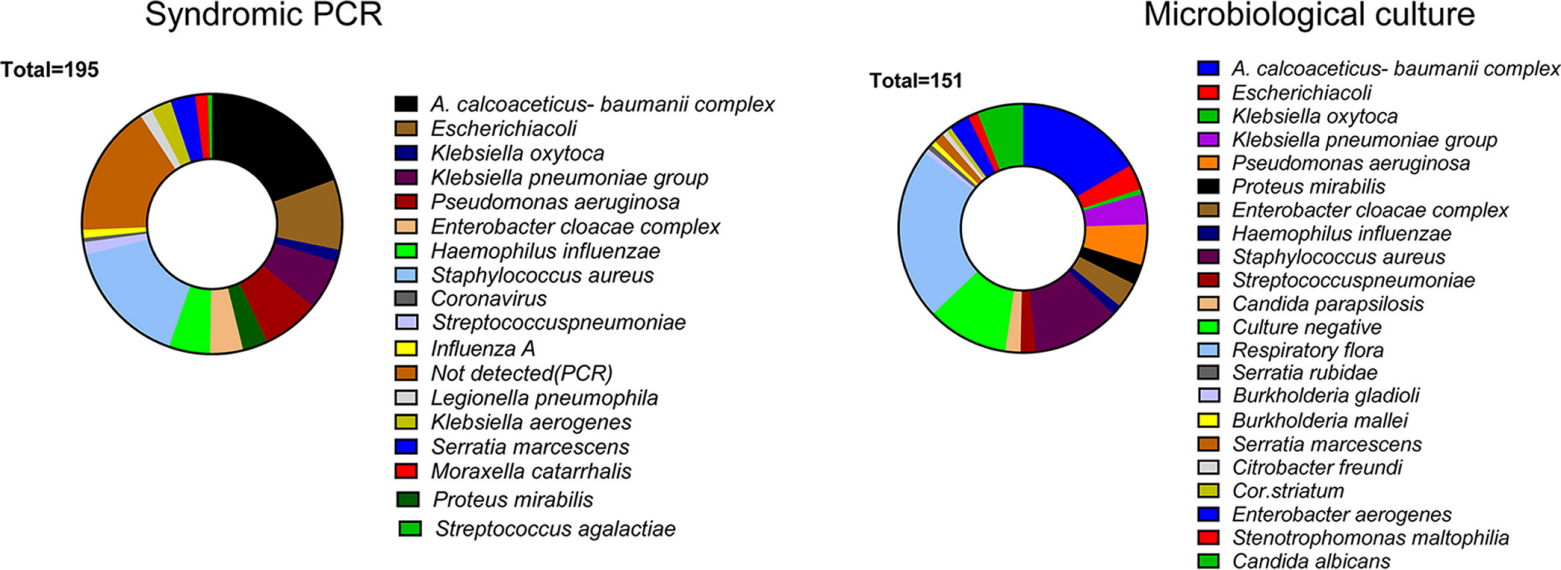
Summary of the results of syndromic PCR with culture of tracheal aspirates or BAL.

**FIG 2:**
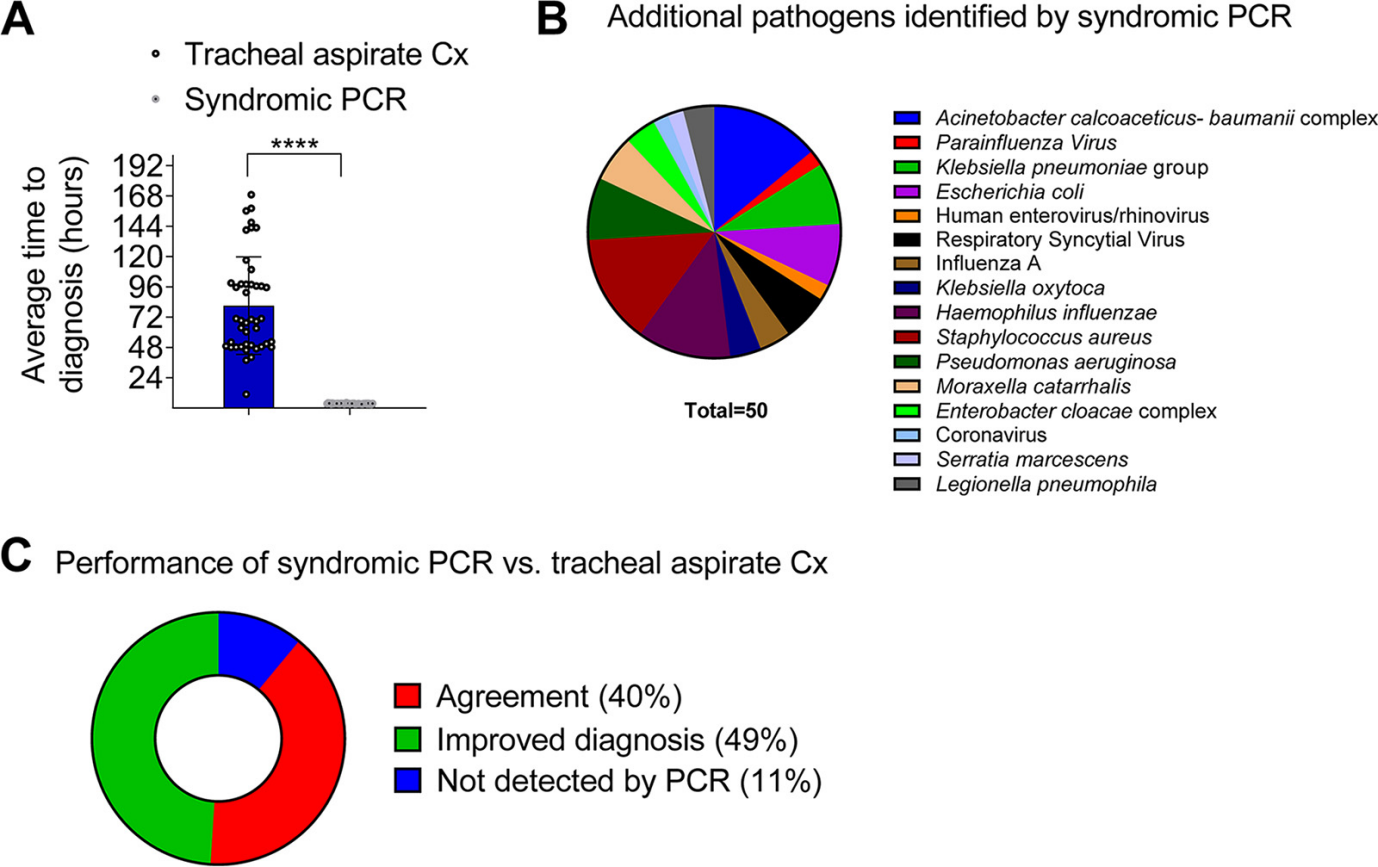
Comparative analysis of performance of syndromic PCR platform versus conventional culture of tracheal aspirates or BAL related to (A) time to diagnosis, (B) new pathogens identified (C) performance in diagnosis.

The causative pathogens of pneumonia in the study (syndromic PCR) and control groups identified by conventional cultures are shown in Table 2. In both groups Gram negative nosocomial pathogens including Acinetobacter, Pseudomonas, *and* Klebsiella species were the main causes of bacterial pneumonia in the ICU. Of interest, there was a trend of increased rates of MDR/XDR causes of pneumonia in the historical control group. Next, we compared the yield of syndromic PCR versus conventional culture of tracheal aspirates in the study group of patients (Fig. 1). We found that the syndromic PCR test (a) was in agreement with culture in 34 patients (40% of cases; Fig. 2 and Table 3) and resulted in correct identification of 29 bacterial pathogens in 19 patients. Accordingly, there was a high degree of concordance (93%; 27/29) between the quantitative culture result (CFU/mL) and the reported semi-quantitative PCR equivalent in these cases (Table 3). Additionally, 44 significant pathogens were exclusively identified by syndromic PCR, which are shown in Table 3. Overall, syndromic PCR provided new information that resulted in improved diagnosis in 42 (49%) of patients with pneumonia (Fig. 2B and C). Nonetheless, in 9 (11%) cases of pneumonia (Fig. 2C), the pathogens were identified only in culture, because the corresponding microbial targets were not included in the syndromic PCR panel; these cases included VAP (*n* = 3) caused by Gram negative pathogens (Stenotrophomonas maltophilia, *Burkolderia* spp., *Serretia* spp.) and 6 co-infections with C. albicans (considered as co-pathogen by the primary physician) (Table 2). Appropriate empirical antimicrobial therapy in bacterial pneumonia not identified by syndromic PCR was guided by Gram stain of the tracheal aspirate.

**TABLE 2:** Results of microbiological cultures of control and syndromic PCR group of patients

Microbial pathogen^*a*^	Control group	Syndromic PCR group
Acinetobacter calcoaceticus *-baumanii complex*	32.5% (*n* = 13)	10% (*n* = 10)
Escherichia coli	10% (*n* = 4)	5% (*n* = 5)
Klebsiella oxytoca	5% (*n* = 2)	1% (*n* = 1)
Klebsiella pneumoniae *group*	10% (*n* = 4)	5% (*n* = 5)
Pseudomonas aeruginosa	22.5% (*n* = 9)	6% (*n* = 6)
Proteus mirabilis	7.5% (*n* = 3)	3% (*n* = 3)
Enterobacter aerogenes	2.5% (*n* = 1)	1% (*n* = 1)
Enterobacter cloacae *complex*	5% (*n* = 2)	5% (*n* = 5)
Haemophilus influenzae	2.5% (*n* = 1)	2% (*n* = 2)
Stenotrophomonas maltophilia	7.5% (*n* = 3)	1% (*n* = 1)
Serratia marcescens	5% (*n* = 2)	0%
Staphylococcus aureus	12.5% (*n* = 5)	11% (*n* = 11)
Streptococcus pneumoniae	5% (*n* = 2)	3% (*n* = 3)
Candida albicans	7.5% (*n* = 3)	6% (*n* = 6)
Candida tropicalis	2.5% (*n* = 1)	0%
Candida parapsilosis	0%	1% (*n* = 1)
*No pathogen identified*	0%	16% (*n* = 16)
*Negative (respiratory tract flora)*	0%	21% (*n* = 21)
*Burkolderia gladioli*	0%	1% (*n* = 1)
*Serratia rubidae*	0%	1% (*n* = 1)
*Burkolderia mallei*	0%	1% (*n* = 1)

a There were 3 cases of bacterial pneumonia caused by Stenotrophomonas maltophilia, *Serratia*, *rubidiae*, *and Bukholderia gladioli* exclusively identified by culture (the relevant targets are not included in the PCR panel). In 6 patients with pneumonia, C. albicans was identified in cultures of tracheal aspirate and considered as co-pathogen by the primary physician (target not included in the PCR panel).

**TABLE 3:** Analysis of additional pathogens identified by syndromic PCR panel^*a*^

Cases	Culture result (CFU/mL)	Syndromic PCR result (corresponding CFU/mL value)^*b*^
1	No Pathogen (culture)	Acinetobacter calcoaceticus*-baumanii complex* (10^6^)
2	Klebsiella pneumoniae *group* (3*10^5^)	Klebsiella pneumoniae *group* (10^6^)*, Parainfluenza Virus*
3	No Pathogen	Klebsiella pneumoniae *group* (10^4^)
4	Acinetobacter calcoaceticus*-baumanii complex* (4*10^7^)	Acinetobacter calcoaceticus*-baumanii complex* (10^5^), Escherichia coli (10^4^)
5	Acinetobacter calcoaceticus*- baumanii complex* (>10^9^)	Acinetobacter calcoaceticus*-baumanii complex* (≥10^7^)*, Parainfluenza Virus*
6	No Pathogen	*Human enterovirus/rhinovirus*
7	No Pathogen	*Respiratory Syncytial Virus*
8	No Pathogen	*Respiratory Syncytial Virus*
9	No Pathogen	*Influenza A, Respiratory Syncytial Virus*
10	No Pathogen	Klebsiella oxytoca (10^5^)
11	No Pathogen	Haemophilus influenzae (10^6^)
12	No Pathogen	Haemophilus influenzae (10^4^)
13	No Pathogen	Staphylococcus aureus (10^4^)
14	No Pathogen	Pseudomonas aeruginosa (10^6^)
15	No Pathogen	Escherichia coli (10^5^)
16	Staphylococcus aureus (4*10^7^), Klebsiella pneumoniae *group* (3*10^5^)*, Burkolderia gladioli* (2*10^6^)	Staphylococcus aureus (≥10^7^), Klebsiella pneumoniae *group* (10^5^), Haemophilus influenzae (10^5^)
17	Staphylococcus aureus (25*10^3^)	Staphylococcus aureus (10^5^), Klebsiella pneumoniae *group* (10^4^)
18	Proteus mirabilis (20*10^7^)	Proteus mirabilis (≥10^7^), Haemophilus influenzae (≥10^7^)
19	Streptococcus pneumoniae (>10^7^)	Streptococcus pneumoniae (≥10^7^), Staphylococcus aureus (10^4^), Streptococcus agalactiae (10^5^)
20	Streptococcus pneumoniae (2*10^5^)	Streptococcus pneumoniae (≥10^7^), Staphylococcus aureus (10^4^)
21	Escherichia coli (>10^7^)	Escherichia coli (≥10^7^), Acinetobacter calcoaceticus*-baumanii complex* (10^4^), Staphylococcus aureus (10^5^), Pseudomonas aeruginosa (10^4^)
22	Enterobacter cloacae *complex* (10^5^)	Enterobacter cloacae *complex* (10^5^), Staphylococcus aureus (10^4^)
23	No Pathogen	Staphylococcus aureus (10^4^)
24	Escherichia coli (6*10^5^)	Escherichia coli (10^6^), Moraxella catarrhalis (10^5^)
25	No Pathogen	Enterobacter cloacae *complex* (10^5^)
26	No Pathogen	Pseudomonas aeruginosa (10^5^)
27	Staphylococcus aureus (>10^7^)	Staphylococcus aureus (≥10^7^), Acinetobacter calcoaceticus*-baumanii complex* (10^5^), Klebsiella pneumoniae *group* (10^5^), Legionella pneumophila
28	No Pathogen	Acinetobacter calcoaceticus*-baumanii complex* (10^5^)
29	Staphylococcus aureus (2*10^7^)	Staphylococcus aureus (≥10^7^)*, Influenza A*
30	Acinetobacter calcoaceticus*-baumanii complex* (>10^9^), Pseudomonas aeruginosa (2*10^7^), Klebsiella pneumoniae *group* (40*10^7^)	Acinetobacter calcoaceticus*-baumanii complex* (≥10^7^), Pseudomonas aeruginosa (10^6^), Klebsiella pneumoniae *group* (10^6^)*, Coronavirus*
31	Escherichia coli (4*10^7^), Proteus mirabilis (80*10^7^)	Escherichia coli (≥10^7^), Proteus mirabilis (≥10^7^), Acinetobacter calcoaceticus*-baumanii complex* (≥10^7^)
32	Klebsiella pneumoniae *group* (5*10^5^), Klebsiella oxytoca (2*10^5^)	Klebsiella pneumoniae *group* (10^6^), Klebsiella oxytoca (10^5^), Staphylococcus aureus (10^5^)
33	No Pathogen	Haemophilus influenzae (10^5^)
34	No Pathogen	Serratia marcescens (10^5^), Klebsiella oxytoca (10^4^)
35	Acinetobacter calcoaceticus*-baumanii complex* (10^9^)	Acinetobacter calcoaceticus*-baumanii complex* (≥10^7^), Escherichia coli (10^5^), Klebsiella pneumoniae *group* (10^5^), Haemophilus influenzae (10^4^), Pseudomonas aeruginosa (≥10^7^)
36	Streptococcus pneumoniae (>10^9^)	Streptococcus pneumoniae (≥10^7^), Haemophilus influenzae (10^6^), Moraxella catarrhalis (10^4^), Proteus mirabilis (10^4^)
37	No Pathogen	Staphylococcus aureus (10^4^), Haemophilus influenzae (10^5^)
38	No Pathogen	Acinetobacter calcoaceticus*-baumanii complex* (10^5^)
39	Candida albicans (10^8^)	Enterobacter cloacae *complex* (10^6^), Escherichia coli (10^4^)
40	No Pathogen	Acinetobacter calcoaceticus*-baumanii complex* (≥10^7^)
41	Haemophilus influenzae (2*10^5^)*, Burkolderia mallei* (10^5^)	Haemophilus influenzae (≥10^7^), Moraxella catarrhalis (≥10^7^)
42	No Pathogen	Legionella pneumophila

a There were 34 cases of bacterial pneumonia with agreement between syndromic PCR and culture results, including 15 cases with no pathogen identified and 19 cases of concordant identification of bacterial pathogens (7 Staphylococcus aureus, 4 Pseudomonas aeruginosa, 5 Acinetobacter calcoaceticus*-baumanii complex*, and 6 bacterial co-infections). Coinfections included 1 case of Enterobacter cloacae *complex* plus Klebsiella pneumoniae *group;* 1 case of Acinetobacter calcoaceticus*-baumanii complex* plus Pseudomonas aeruginosa*;* 1 case of Staphylococcus aureus *plus* Enterobacter cloacae *complex and* Proteus mirabilis*;* 1 case of Staphylococcus aureus *plus* Pseudomonas aeruginosa*;* 1 case of Staphylococcus aureus *plus* Enterobacter cloacae *complex;* and 1 case of Escherichia coli, Klebsiella aerogenes. In all 19 cases of bacterial pneumonia with concordant identification of the pathogen, there was excellent agreement (38/40, 95%) between the quantitative culture result reported in CFU/mL and the semi-quantitative result of the syndromic PCR reported as CFU/mL equivalent gene copy numbers. In two cases syndromic PCR underestimated the bacterial burden compared to microbiological culture (Enterobacter aerogenes 10^4^ vs. 2 × 10^7^ CFU/mL; Pseudomonas aeruginosa 10^4^ vs. 2 × 10^7^ CFU/mL).

b The syndromic PCR panel identified 12 cases with resistance genes; 9 of them (5 cases of Staphylococcus aureus mecA/C and MREJ, 2 cases of Escherichia coli CTX-M, 1 case of Escherichia coli KPC and 1 case of Klebsiella pneumonia KPC) were in agreement with the phenotype in susceptibility testing of the isolate. In 3 additional cases PCR detected additional resistance genes (Klebsiella pneumonia CTX-M, Staphylococcus aureus mecA/C and MREJ, Escherichia coli VIM). Detection of resistance genes was not considered as an indication of improved diagnosis, because in several cases of MDR/XDR pathogens with complex resistance mechanisms (e.g., Pseudomonas, Acinetobacter) the resistance phenotype was not identified by syndromic PCR.

The syndromic PCR panel correctly identified resistance genes in 12 cases of bacterial pneumonia caused by S. aureus (*n* = 6), E. coli (*n* = 4), and Klebsiella spp. (*n* = 2), in agreement with the phenotype of susceptibility testing of the corresponding isolates (Table 3). However, the syndromic PCR failed to detect resistance phenotype in other MDR/XDR pathogens including Pseudomonas and Acinetobacter, which display complex underlying resistance mechanisms. Collectively, in comparison the conventional cultures PCR syndromic test resulted in sharp decrease in time to diagnosis (Fig. 2A) and provided new microbiological information in almost half of the cases of pneumonia (49%) (Fig. 2B and C).

**FIG 3:**
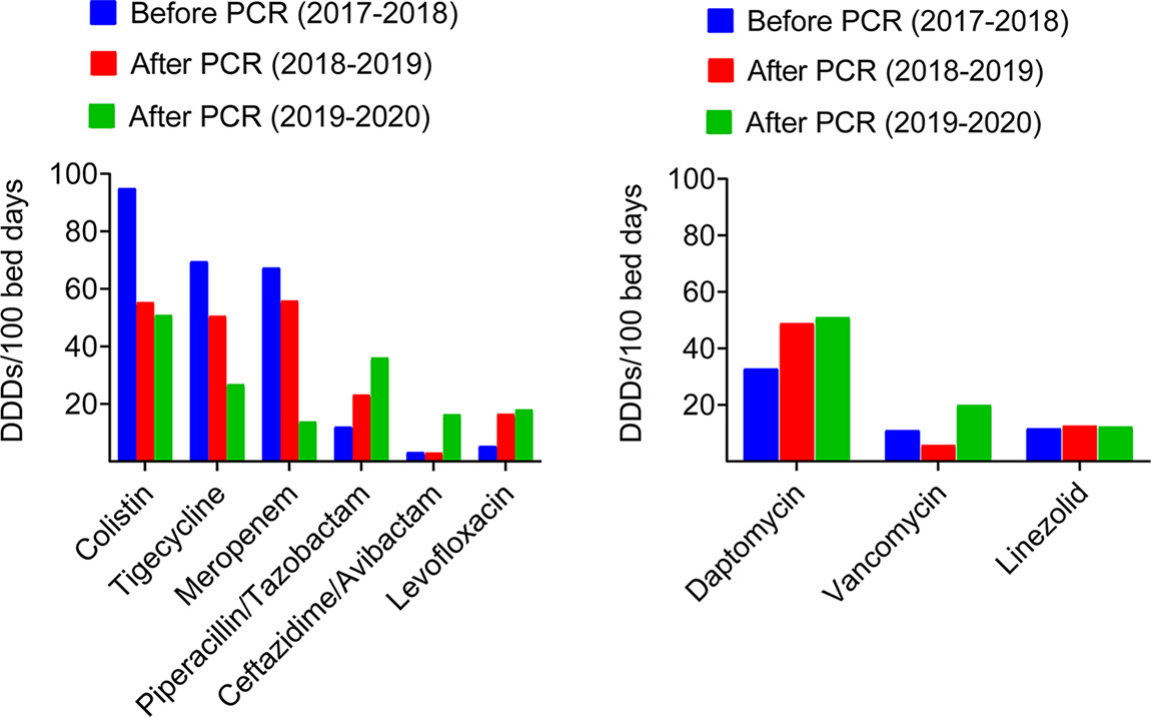
Trends in consumption of antibiotics against Gram negative (left panel) and Gram positive (right panel) bacterial pathogens in control and syndromic PCR group of patients over the study period.

Next, we assessed the impact of syndromic PCR on antibiotic usage by comparing antimicrobial consumption (expressed in DDD/100 BD) in the study group versus the historical control group (Table 4 and Fig. 3). Of interest, we noticed a significant reduction in use of antimicrobials targeting MDR/XDR pathogens, including colistin, tigecycline, and carbapenems (Table 4 and Fig. 3). In parallel, we found a significant increase in use of b-lactam/b-lactamase inhibitors (e.g., ampicillin/sulbactam, piperacillin/sulbactam, and ceftazidime-avibactam) and levofloxacin following implementation of syndromic PCR, which is consistent with antimicrobial de-escalation from broad-spectrum to narrow spectrum antimicrobial therapy. Importantly, the effect of syndromic PCR on reduced consumption of the aforementioned antibiotics was evident across the years of the study (Fig. 3).

**TABLE 4:** Comparative analysis of antibiotic consumption in control and syndromic PCR group of patients expressed as DDD/100 bed days (BD) values

Antibiotic	Control group (DDDs/100BD)	Syndromic PCR group (DDDs/100BD)	*P* value
Ampicillin/sulbactam	6.7	13.22	0.1177
Piperacillin tazobactam^*a*^	11.96	33.02	0.0003
Ceftazidime–Avibactam^*b*^	3.14	13.23	0.0091
Ceftazidime		2.6	
Ceftriaxone	2.49	0.52	0.9999
Cefepime	14.97	16.63	0.5875
Meropenem	67.22	23.61	<0.0001
Levofloxacine	5.3	17.64	0.0027
Ciprofloxacine	4.99	2.67	0.728
Colistin^*c*^	94.86	51.91	0.0011
Vancomycin	10.91	16.63	0.1772
Tigecycline	69.44	32.36	0.0008
Gentamycin	5.13	1.14	0.2203
Daptomycin	32.74	50.49	0.0137
Amikacin	2.08	3.13	0.6718
Clindamycin	0.69	0.65	0.9999
Linezolid	11.54	12.37	0.8373
Trimethoprime sulfomethoxazol		12.18	NA^*d*^
Metronidazol	4.44	2.69	0.9999
Rifampicine	1.25	1.41	0.9999
Itraconazole	0.21	4.98	0.0234
Fluconazole	0.73	1.3	0.9999
Isavuconazole		1.62	NA
Amphotericin B		4.65	NA
Micafungin	2.29	16.41	0.0004
Anidulafungin	4.99	5.73	0.7639
Oseltamivir		1.75	NA
Ceftolozan tazobactam	1.04	1.56	0.6057
Tobramycin		0.65	NA
Acyclovir		1.06	NA
Azithromycin		0.26	NA
Isoniazid		0.85	NA
Ceftraolin fosamil		2.34	NA
Voriconazole		0.13	NA
Moxifloxacin		0.13	NA

a There were 15 cases of pneumonia in the control group and 33 cases of pneumonia in the PCR group that received appropriate therapy with piperacillin-tazobactam.

b There were 2/40 and 19/79 patients with pneumonia in the control and syndromic PCR groups who received Ceftazidime-Avibactam, respectively. These included 1 case of carbapenem resistant Klebsiella pneumoniae in the control group, 8 cases of Acinetobacter baumannii (2 PDR and 6 XDR) and 2 cases of carbapenem resistant (KPC) Klebsiella pneumoniae in the PCR group.

c Examples of de-escalation due to discontinuation of colistin in VAP include (a) a patient with MRSA pneumonia (S. aureus, detection of mecA/C-MREJ resistance genes) who received empirical antimicrobial therapy with colistin plus piperacillin-tazobactam and following PCR result the treatment was modified to linezolid/cefepime (b) a patient with pneumonia caused by Enterobacter cloacae on empirical therapy with colistin plus piperacillin-tazobactam who received piperacillin-tazobactam following the PCR result, and (c) a patient of mixed infection by Enterobacter aerogenes plus Klebsiella pneumoniae on empirical therapy with meropenem/colistin who received piperacillin-tazobactam following the PCR result. In addition to de-escalation, targeted antimicrobial therapy was started upfront based on the PCR report in several cases of pneumonia (e.g., influenza, *Legionella*).

d NA, not applicable.

## DISCUSSION

Previous studies demonstrated an excellent performance of syndromic PCR panel in diagnosis of the causative agent of pneumonia compared to conventional microbiological cultures (14, 15). Of interest, these studies including patients with CAP (14, 15), HAP, or VAP of different disease severity and provided the rationale of use of syndromic PCR as a complementary tool for de-escalation of antimicrobial therapy within the prospect of a dedicated antimicrobial stewardship program (15). However, these studies included a limited number of MDR or XDR pathogens, and a group of patients who did not receive Colistin, Tigecycline and other last resource antimicrobial therapies. Therefore, the feasibility and impact of syndromic PCR in a setting of high prevalence of MDR pathogens has not been previously evaluated.

Herein, we employed a study in ICU patients hospitalized with different types of severe pneumonia (CAP, HAP, VAP) with a need for mechanical ventilation. The majority of these patients (> 60%) had VAP due to MDR and XDR bacterial pathogens. In contrast to previous studies, we employed a historical control group of patients with pneumonia hospitalized in the ICU during the previous year of the study. Importantly, the control group had comparable clinical and microbiological features with the PCR syndromic group, which allowed to study the impact of the diagnostic platform on antibiotic consumption rates in a relatively unbiased approach. Furthermore, the choice of antimicrobial therapy guided by the PCR result was a decision made by the primary physician (intensivist) following discussion with the primary investigators, rather than by an expert committee (15), a practice that reflect on the real use of this diagnostic platform on the daily hospital care. The results of the study clearly validate the use of syndromic PCR in the ICU setting, particularly in the setting of high rates of antimicrobial resistance as a complementary intervention to reduce selection pressure of MDR/XDR pathogens and reduce toxicity associated with the use of broad spectrum empirical antimicrobial therapies. Importantly, comprehensive assessment of the clinical utility of a new syndromic PCR test should evaluate the impact on additional parameters, including length of hospital stay and cost of patient care. Additionally, implementation of antibiotic stewardship substantially improves cost-effectiveness of rapid molecular diagnostic tests in infectious diseases (16) and should be evaluated in future studies on syndromic PCR panel for pneumonia.

Limitations of the study include its retrospective nature, possible changes in infection control and antimicrobial therapy strategies which have not been captured by the investigators of the study (e.g., the impact of the feedback of clinical microbiologist on decisions in empirical antibiotic therapy is difficult to measure), the single center nature of the study and the relatively limited numbers of patients enrolled. Furthermore, the lack of certain nosocomial pathogens (e.g., *Stenotrophomonas*) in the molecular platform requires increased vigilance and the need for complementarity with other diagnostic procedures (e.g., detection of a broad microbial target such as 16S RNA to indicate the presence of a non-identified pathogen) to ensure optimal patient care. The clinical relevance of detection of microbial causes of VAP at relatively low copy numbers (e.g., corresponding value of 10^4^ CFU/mL) exclusively by syndromic PCR requires validation in future studies. While early report of resistance mechanism in very useful for targeted antibiotic therapy (e.g., KPC versus NDM Klebsiella spp.), the PCR panel does not capture complex resistance mechanisms in certain nosocomial pathogens (e.g., Pseudomonas spp., Acinetobacter) leading to potential misguidance of therapy. Therefore, microbiological cultures remain the gold standard in diagnosis and guidance on targeted therapy in severe bacterial pneumonia. Nonetheless, molecular syndromic platforms should be regarded as important adjunct diagnostic tools for antibiotic stewardship and timely diagnosis of non-culturable pathogens (e.g., viruses). Collectively, our study illustrates that the PCR syndromic approach is a useful tool in the management of pneumonia in the ICU, especially in the nosocomial setting of high rates of MDR/XDR nosocomial pathogens.

## MATERIALS AND METHODS

### Study participants.

Between June 2018 and June 2020 all patients admitted the ICU of University Hospital of Heraklion, Crete with severe CAP/HAP or those who developed VAP during ICU stay were eligible for enrollment to the study. The following criteria were required for patient inclusion: (1) age ≥ 18 years, (2) presence of clinical and radiological criteria for pneumonia according to the IDSA guidelines: new lung infiltrate on a chest X-ray and evidence that the infiltrate was of an infectious origin, i.e., at least two of three clinical features (fever greater than 38°C, leukocytosis or leukopenia, and purulent secretions), and (3) severe respiratory failure requiring ICU admission and mechanical ventilation. Criteria for pneumonia were retrospectively evaluated by two clinical investigators (K.V., and D.G.). Severe CAP or HAP requiring ICU admission, and VAP were included. HAP was defined as pneumonia occurring 48 h or more after admission, which was not incubating at the time of admission and not associated with mechanical ventilation (11, 12). VAP referred to pneumonia occurring > 48 h after endotracheal intubation (11). CAP included all episodes of pneumonia acquired outside of the hospital setting. The presence of immunodeficiency, malignancy or other co-morbidity was not considered as an exclusion criterion. All intubations were preformed according to “American Journal of respiratory and critical care medicine” (13). Endotracheal aspirates were obtained from all the patients at the time of clinical indication of pneumonia and tested simultaneously, using conventional microbiological techniques and the PCR BioFire FilmArray Pneumonia Panel (bioMérieux S.A., France). The following targets are included in the test: Acinetobacter calcoaceticus*-baumannii complex*, Enterobacter cloacae, Escherichia coli, Haemophilus influenzae, Klebsiella aerogenes, Klebsiella oxytoca, Klebsiella pneumoniae *group*, Moraxella catarrhalis, Proteus spp., Pseudomonas aeruginosa, Serratia marcescens, Staphylococcus aureus, Streptococcus agalactiae, Streptococcus pneumoniae, Streptococcus pyogenes, *Chlamydia pneumoniae, Legionella pneumophila, Mycoplasma pneumoniae, Adenovirus, Coronavirus, Human metapneumovirus, Human rhinovirus/enterovirus, Influenza A vírus, Influenza B vírus, Parainfluenza virus, Respiratory syncytial virus*, CTX-M, KPC, NDM, Oxa48-like, VIM, IMP, mecA/mecC, and MREJ.

Exclusion criteria included (i) ICU patients with fever of another etiology, (ii) colonization of the lung or ventilator associated tracheobronchitis (VAT), (iii) patient already enrolled in the study, and (iv) patients on palliative care.

### Microbiology methods.

Endotracheal aspirate (EA) samples with <10 epithelial cells per low power field on Gram-stained smears were acceptable for culture (3). The respiratory samples (EA and bronchoalveolar lavage (BAL) fluid) were quantitatively cultured for aerobic microorganisms using chocolate agar, sheep blood agar, and MacConkey agar plates. Plates were incubated overnight in a 5% CO_2_ atmosphere at 35°C for 48 h. Colonies were then counted and bacterial concentrations (CFU/mL) were calculated. Diagnostic thresholds of ≥ 10^4^ CFU/mL and ≥ 10^5^ CFU/mL were adopted for BAL cultures and EA, respectively; bacteria grown in numbers < 10^4^ CFU/mL were not reported as a relevant cause of pneumonia (3). Pure cultures and dominant pathogens were identified by the VITEK2 system (bioMérieux). Diagnostic tests for viruses and atypical bacteria were not conducted routinely. Respiratory samples were simultaneously tested upon receipt with the BioFire FilmArray Pneumonia Panel according to the manufacturer’s instructions directly on native respiratory samples.

### Clinical data.

Clinical and demographical characteristics were retrospectively obtained from the electronic medical records of each patient. Investigators collected demographic characteristics (age, gender) and medical data including comorbidities, classification of pneumonia (VAP, HAP, or CAP), severity scores, antibiotics prescribed, and outcome. For each episode, the result of the syndromic PCR was presented to the primary physician on call (intensivist) and the primary investigators of the study (D.S., G.C., K.V.) in real time. The clinical microbiologist in charge of the study (D.S.) provided explanation on the results of the syndromic PCR test to the primary physician, including discussion on antibiotic choices based on detected pathogen and the results of the genetic markers of antimicrobial resistance. Detailed information on the susceptibility of the predominant causes of VAP in the ICU (e.g., Pseudomonas, Klebsiella spp.) in the year before the study was provided to all participating physicians. The primary physician decided on the most appropriate antimicrobial therapy for each pneumonia episode.

### Endpoints.

Primary aim of the study was to evaluate the rationale for broad use of BIOFIRE FILMARRAY Pneumonia Panel plus in routine management of severe CAP/HAP and VAP in hospitals with high rates of MDR and pan-drug resistant (PDR) bacterial pathogens. To address this endpoint, we performed comparative analysis of the rates of antimicrobial consumption in ICU patients with pneumonia the year before (2018; historical control group) and over the study period (2018–2020). Rates of antibiotic consumption were expressed as defined daily dose (DDD) per 100 bed days (14).

Additionally, the following measures were assessed: The accuracy of multiplex PCR compared to routine microbiological diagnosis, the decrease in time to diagnosis associated with the use of multiplex PCR; the impact of molecular diagnosis on establishment of etiological diagnosis of severe pneumonia and VAP; the effect of rapid molecular diagnostic result on the outcome of patients, defined by days of ICU hospitalization and overall mortality.

### Statistical analysis.

All data were anonymously collected and stored on a secured database. Two-sided unpaired Student's *t* test was used for statistical comparisons of continuous variables between the two groups. Fisher’s exact test was used for analysis of categorical variables. *P < *0.05 was considered statistically significant. Analysis was performed using the GraphPad Prism software (version VII). The study was approved by the Ethics Committee of the University of Crete (IRB approval # *58/16.07.2020).*

The data sets used during the current study are available from the corresponding author on reasonable request.
